# Regulation of a vacuolar proton-pumping P-ATPase MdPH5 by MdMYB73 and its role in malate accumulation and vacuolar acidification

**DOI:** 10.1007/s42994-023-00115-7

**Published:** 2023-09-22

**Authors:** Xiao-Yu Huang, Ying Xiang, Yu-Wen Zhao, Chu-Kun Wang, Jia-Hui Wang, Wen-Yan Wang, Xiao-Long Liu, Quan Sun, Da-Gang Hu

**Affiliations:** National Research Center for Apple Engineering and Technology, Shandong Collaborative Innovation Center of Fruit and Vegetable Quality and Efficient Production, College of Horticulture Science and Engineering, Shandong Agricultural University, Tai’an, 271018 Shandong China

**Keywords:** Apple, Malate accumulation, P-ATPase, MdPH5, MdMYB73

## Abstract

As the main organic acid in fruits, malate is produced in the cytoplasm and is then transported into the vacuole. It accumulates by vacuolar proton pumps, transporters, and channels, affecting the taste and flavor of fruits. Among the three types of proton pumps (V-ATPases, V-PPases, and P-ATPases), the P-ATPases play an important role in the transport of malate into vacuoles. In this study, the transcriptome data, collected at different stages after blooming and during storage, were analyzed and the results demonstrated that the expression of *MdPH5*, a vacuolar proton-pumping P-ATPase, was associated with both pre- and post-harvest malate contents. Moreover, MdPH5 is localized at the tonoplast and regulates malate accumulation and vacuolar pH. In addition, MdMYB73, an upstream MYB transcription factor of *MdPH5*, directly binds to its promoter, thereby transcriptionally activating its expression and enhancing its activity. In this way, MdMYB73 can also affect malate accumulation and vacuolar pH. Overall, this study clarifies how MdMYB73 and MdPH5 act to regulate vacuolar malate transport systems, thereby affecting malate accumulation and vacuolar pH.

**Supplementary Information:**

The online version contains supplementary material available at 10.1007/s42994-023-00115-7.

## Introduction

Organic acids affect the acidity of fleshy fruits and play an important role in the regulation of osmotic pressure, pH homeostasis, stress resistance, and sensory quality of fruits (Huang et al. 2021). Malate is one of the most important organic acids in fruits, and accounts for 90% of total organic acids in apple. Malate can generate ATP, resist oxidation, and relieve oxidative stress (Etienne et al. 2013). Additionally, it can also relieve angiosclerosis, facilitate absorption of calcium, iron, and other elements, and stimulate secretion by digestive glands.

In fruit cells, malate is mainly synthesized in the cytoplasm, via phosphoenolpyruvate carboxylation (PEP), and is typically catalyzed by phosphoenolpyruvate carboxylase (PEPC) and malate dehydrogenase (MDH) (Sweetman et al. 2009). Malate is then transported into vacuoles for storage. Although malate accumulation in cells is controlled by both metabolism and vacuolar storage, the transport process from  the cytoplasm into vacuoles may have a determining effect on malate accumulation. Therefore, it is necessary to thoroughly investigate this transport process. As a matter of fact, this process involves multiple vacuolar transporters, ion channels and carriers, among which vacuolar transporters and channels play a dominant role in the transport of organic acids, such as the tonoplast dicarboxylate transporter (tDT) (Emmerlich et al. 2003) and an aluminium-activated malate transporter (ALMT) (such as ALMT6 and ALMT9/Ma1) (Emmerlich et al. 2003; Meyer et al. 2011; Cohen et al. 2014; Martinoia 2018). In addition, some proton pumps on the tonoplast also play a key role in this process by transporting H^+^ into vacuoles and acidifying vacuoles. In this way, the proton electrochemical gradients that serve as the driving force for the transport of malate into vacuoles are generated.

To date, three types of proton pumps, including vacuolar H^+^-ATPase (V-ATPase), vacuolar H^+^-PPase (V-PPase), and vacuolar P-type ATPase (P-ATPase), have been identified in fruits (Hurth et al. 2005; Kovermann et al. 2007; Martinoia et al. 2007). As a novel proton pump thoroughly investigated in recent years, the P-ATPase was first identified in petunia and may be associated with vacuole acidification. In plants, P-ATPase consists of five major evolutionarily related subfamilies (P1-P5). Among them, ATPases in the P3 subfamily are responsible for energizing the electrochemical gradient serving as the driving force of secondary transport (Pedersen et al. 2012). The P-type ATPases (PhPH5 and PhPH1) found in petunia are involved in the vacuolar acidification of petal cells, whereas only PhPH5 shows independent proton transport activity (Verweij et al. 2008; Faraco et al. 2014; Li et al. 2016). Moreover, their homologous genes were also identified in fruits. In citrus, the homologous gene, *CitPH5*, is closely related to the generation of super-acidified fruit (Strazzer et al. 2019). In pear, PbPH5 is localized to the vacuolar membrane and can promote malate accumulation in fruits (Song et al. 2022). As a homologue of *PhPH5* in apple, *MdPH5* is slightly upregulated in transgenic MdMYB73 callus, which is responsible for malate accumulation and vacuolar acidification (Hu et al. 2017), suggesting that *MdPH5* may also be associated with malate accumulation in apple. Nevertheless, the specific function of *MdPH5* in apple fruits remains unclear.

The regulation of organic acid transporters and proton pumps involves complex gene regulatory networks. Indeed, transcriptional regulation is one of the most common events. Among them, MYB transcription factor (TF) families comprise plant-specific R2R3-MYB TFs, many of which are involved in the transport of organic acids by activating or inhibiting gene expression of transporters and proton pumps. Remarkably, MdMYB1, MdMYB44, and MdMYB73, which are present in apple, can affect the transcriptional activity of malate transporters and proton pumps to regulate malate accumulation and vacuolar acidification (Hu et al. 2016, 2017; Jia et al. 2021). *CrMYB73*, which is homologous to *MdMYB73*, leads to increased citrate accumulation in citrus plants, whereas its downstream target genes remain unclear (Li et al. 2015). In addition, PhPH4 in petunia is an R2R3-MYB TF that plays a similar role to VvMYB5a and VvMYB5b in grape for the regulation of citrate accumulation. Specifically, they both can activate expression of the downstream genes *PH1* and *PH5*, thus acidifying vacuoles (Cavallini et al. 2014; Kasajima et al. 2016; Amato et al. 2019). In soybean petals, GmPH4, which is an R2R3-MYB TF, is also involved in vacuolar acidification as it directly regulates the expression of *GmPH5*, a P_3A_-type ATPase gene (Sundaramoorthy et al. 2020). However, the specific regulators of *MdPH5* in apple have not yet been identified.

In this study, the role of *MdPH5* in regulating malate accumulation and vacuolar acidification in apple was investigated and its transcriptional regulation by MYB TF MdMYB73 was verified. This study provides a reference for understanding the molecular factors involved in fruit quality.

## Materials and methods

### Plant materials and growth conditions

‘*Orin*’ apple calli obtained from young embryos were subcultured at 3-week intervals on MS medium supplemented with 1.5 mg/L 2,4-dichlorophenoxyacetic acid (2,4-D) and 0.4 mg/L 6-benzylaminopurine (6-BA) at 25 °C, in the dark. Subsequently, these calli were subcultured, at half-month intervals, three times before being used for further studies.

‘*Royal Gala*’ apples were collected at 41, 70, 94, 128 days post-flowering. ‘*Royal Gala*’ apple were collected at 120 days post-flowering and stored in air-conditioned tanks. Batch of apples of similar size, color, maturity, disease-free, insect-free, and without mechanical damage were selected for storage for 120 days, and samples were taken every 30 days. These apples were frozen in liquid nitrogen.

### Bioinformatics analysis of the *MdPH5* gene

The basic information of the *MdPH5* sequence was obtained from the NCBI database (https://www.ncbi.nlm.nih.gov/). The MdPH5 secondary structure prediction was adopted from SOPMA (https://npsaprabi.ibcp.fr/cgi-bin/npsa_automat.pl?page=npsa_sopma.html).

### Phylogenetic analysis of PH5 proteins

The MEGA_X based on the neighbor-joining method and bootstrap analysis with 1000 replications was used to construct the phylogenetic tree of MdPH5 and *Arabidopsis thaliana* P_3A_ subfamily.

### Analysis of the *MdPH5* promoter

The *cis* element in the *MdPH5* promoter (1500 bp upstream of the transcription initiation site) was analyzed with the online software PlantCARE (http://bioinformatics.psb.ugent.be/webtools/plantcare/html/).

### Construction of the *MdPH5* gene expression vector and genetic transformation of apple calli

The ORF sequence of *MdPH5* was cloned into pRI-101 to obtain the overexpression vector, and the non-conserved regions of *MdPH5*’s ORF were reversely cloned into pRI-101 to obtain the antisense vector. Transgenic apple calli were obtained by *Agrobacterium*-mediated transformation.

### Quantitative real-time-PCR (RT-qPCR) analysis

Plant RNA was extracted with an RNA extraction kit (TIANGEN, Beijing, China) and single-stranded cDNA was obtained with a reverse transcription kit (TaKaRa, Shiga, Japan). The RT-qPCR analyses were executed with three biological and technical replications to test the expression levels of *MdPH5*, which were performed with the methods as described by Hu et al. (2016). The quantitative analysis of results used the 2^−∆∆CT^ method.

### Analysis of subcellular localization

The full-length coding sequences of *MdPH5* were fused to the GFP protein, to construct the fusion expression vector 35S:*MdPH5-GFP*, and the resulting plasmid was transformed into *Agrobacterium* strain LBA3101. The constructed vector was injected into tobacco (*Nicotiana benthamiana*) epidermal cells and cultured in the dark for 3 days. The AtCBL-red fluorescent protein (RFP) was used as a vacuolar membrane marker (Ma et al. 2019) and was co-transformed with 35S:*MdPH5-GFP*. Fluorescence images were obtained at 488 nm with a high-resolution laser confocal microscope (LSM880, Zeiss, Meta, Jena, Germany).

### Viral vector-mediated transient expression in apple skins

Apple skin injection assays were performed as described previously. MdPH5-TRV (TRV1 + MdPH5-TRV2) was suppression expression vector. MdPH5-IL60 (IL60-1 + MdPH5-IL60-2) was overexpression vector, MdMYB73-IL60 and so on. IL60 (IL60-1 + IL60-2) and TRV (TRV1 + TRV2) were empty vectors and used as references. MdPH5-TRV2 + MdMYB73-IL60 was mixed with MdPH5-TRV (TRV1 + MdPH5-TRV2) and MdMYB73-IL60 (IL60-1 + MdMYB73-IL60-2) and injected into apple fruit. IL60 + TRV (IL60-1 + IL60-2 + TRV1 + TRV2) was empty vector and used as reference.

### Measurement of vacuolar pH

Isolation of protoplasts from apple calli and measurement of vacuolar pH were carried out, as previously described by Hu et al. (2016). The vacuolar pH was detected with the cell-permeant and pH-sensitive fluorescent dye, 2′,7′-bis(2-carboxyethyl)-5(6)-carboxyfluorescein (BCECF)-AM, while vacuolar pH was quantified by the ratio of pH-dependent (488 nm) and pH-independent (458 nm) excitation wavelengths from a calibration curve, and ratio images were generated with the ion concentration tool of Zeiss LSM confocal software.

### Determination of malate content

Malate content was measured by high-performance liquid chromatography, as previously described by Hu et al. (2016).

### EMSA

EMSA was conducted according to Xie et al. (2012). *MdMYB73* was cloned into the expression vector pGEX4T-1. The MdMYB73-GST recombinant protein was expressed in *Escherichia coli* strain BL21. An oligonucleotide probe of the MdMYB73 promoter was labeled using an EMSA probe biotin labeling kit (Beyotime) according to the manufacturer’s instructions. The recombined protein of MdMYB73-GST was incubated with 10 × binding buffer, 1 μg/μL poly (dI-dC), and 400 fmol of biotin-labeled double-stranded binding consensus oligonucleotides (total volume 20 μL) using a LightShift Chemiluminescent EMSA Kit (Thermo Scientific). The binding reaction was performed at room temperature for 20 min. The DNA–protein complexes were separated on 6.5% non-denaturing polyacrylamide gels, electrotransferred, and detected following the manufacturer’s instructions. The binding specificity was also examined by competition with a fold excess of unlabeled oligonucleotides.

### LUC assay

The apple *MdPH5* promoter fragments were amplified by PCR and cloned into the pGreenII 0800-LUC vector to construct the LUC reporter vector (MdPH5pro-LUC). The full-length coding sequences of *MdMYB73* were cloned into the effector vector pGreenII 62-SK. Individual combinations of reporter vectors and effector vectors were transformed into *Agrobacterium* strain GV3101 cells alongside the pSOUP vector. The *Agrobacterium* strains were used to tobacco (*Nicotiana benthamiana*) epidermal cells. A live-imaging apparatus was used to measure luminescence after 2 days.

### Chromatin immunoprecipitation qPCR analysis

35S:MdMYB73-GFP and 35S::GFP transgenic apple cultures were used for the ChIP-qPCR analysis. The anti-GFP antibody (Beyotime) was used for chromatin immunoprecipitation (ChIP), as described by Xie et al. (2012). The resultant samples were used as templates for qPCR assay.

### Data presentation and statistical analysis

The data obtained in this study were analyzed by DPS Software (Enfield, UK), with *P* < 0.05 considered as indicative of significant differences.

## Results

### Analysis of transcriptome and multiple metabolites in apple fruit at different developmental stages

**Fig. 1:**
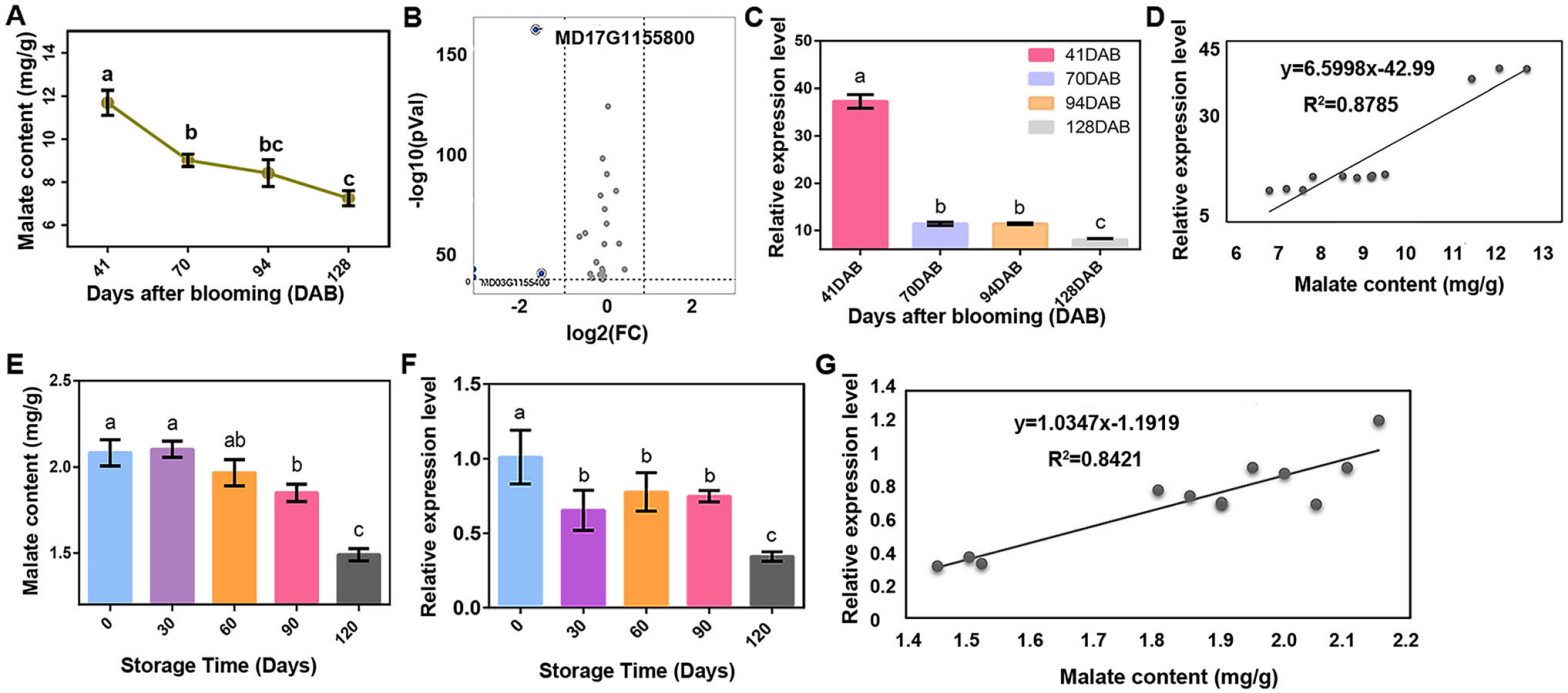
Correlation of *MdPH5* expression with malate content in apple fruit during fruit development and storage. **A** Malate contents in apple fruit on different DAB. **B** Volcanic maps of malate-related genes in the 41 DAB vs. 70 DAB group. **C** The expression levels of *MdPH5* on different DAB. **D** Correlation of the *MdPH5* expression with malate content in apple at different developmental stages. **E** Malate contents at different storage periods. **F** Expression of *MdPH5* in different storage periods determined by RT-qPCR. **G** Correlation of *MdPH5* expression with malate content at different storage periods. Herein, letters indicate significant differences (*P* < 0.05) among the culture media for the various parameters, based on the two-way ANOVA, followed by Duncan’s multiple range test. Bars are SE (*n* = 3)

To clarify the trends of different metabolites in apple fruits after flowering, the contents of major carbohydrates and malate in apple fruits, 41, 70, 94, and 128 days after blooming (DAB), were determined by gas chromatography–mass spectrometry (Fig. S1A). These results showed that the malate content decreased gradually and regularly after flowering (Fig. 1A). The levels of both galactose and sorbitol were minimized by 70 DAB, whereas the level of starch was maximized by 70 DAB (Fig. S1B).

A total of 12 samples, collected at 4 stages were investigated, using transcriptome analysis. The number of upregulated and downregulated genes, in 41 DAB vs. 70 DAB, 70 DAB vs. 94 DAB, and 94 DAB vs. 128 DAB, were 1746 and 4426, 1289 and 1876, and 2435 and 4939, respectively (Fig. S2A). Figures S2B and S2C are Venn diagrams reflecting the overlap of differential genes in different cases. As observed, the malate content decreased regularly and the expression of genes that might be associated with malate showed changes according to the transcriptomic data, as shown in the heat maps. Figure S2D shows the expression levels of 32 malate-related genes, at different developmental stages.

According to the heat maps of the expression levels of malate-related genes, the malate content decreased significantly from 41 to 71 DAB. As observed, genes such as *MD03G1155400*, *MD13G1044200*, and *MD17G1155800* were significantly downregulated (Fig. S3A). To visualize changes of malate-related genes and identify genes with the highest correlation with malate, three volcano maps (41 DAB vs. 70 DAB, 41 DAB vs. 94 DAB, and 41 DAB vs. 128 DAB) were developed. The number of downregulated genes in the volcano maps were 4, 6, and 6, respectively. Among these genes, *MD17G1155800* was the only malate-related gene present in all data sets (Fig. 1B; Fig. S3B, C). As malate was significantly downregulated in 41 DAB vs. 70 DAB, 41 DAB vs. 94 DAB, and 41 DAB vs. 128 DAB, MD17G1155800 may be positively correlated with malate. It has been reported that Md17G1155800 is a P-type proton pump (MdPH5), and its homologous gene, *PbPH5*, in petunia can acidify vacuoles and change petal color (Faraco et al 2014).

### Correlation of *MdPH5* expression with malate content during apple fruit ripening and post-harvest

To further clarify the role of *MdPH5* in apple, the level of *MdPH5* expression in the transcriptome dataset was monitored. The results showed that its expression level decreased (Fig. 1C). Correlation analysis of *MdPH5* and different carbohydrates and acid substances revealed that *MdPH5* expression was highly correlated with malate (*R*^2^ = 0.8787; *P* < 0.05) (Fig. 1D). Unlike for the acids, the contents of galactose, starch, sorbitol, maltose, and glucose had low correlations with the expression of *MdPH5* (Fig. S4A–E). Although the correlation between *MdPH5* expression and fructose content was relatively high (*R*^2^ = 0.8159) (Fig. S4F), unlike the correlation between *MdPH5* expression and malate content, the correlation between them was negative. These results indicated that *MdPH5* is positively correlated with malate accumulation in apple plants.

To clarify the impacts of *MdPH5* on malate content, during storage, Gala apple fruit stored for 0, 30, 60, 90, and 120 days were employed as test samples. These results demonstrated that the malate content decreased gradually with the extension of storage (Fig. 1E). Subsequently, RT-qPCR analysis of samples, at different post-harvest stages, showed that the expression of *MdPH5* also decreased gradually with increasing storage time (Fig. 1F), indicating a positive correlation of *MdPH5* expression with the malate content (*R*^2^ = 0.8421; *P* < 0.05) (Fig. 1G).

### Bioinformatics analysis and subcellular localization of MdPH5 protein

**Fig. 2:**
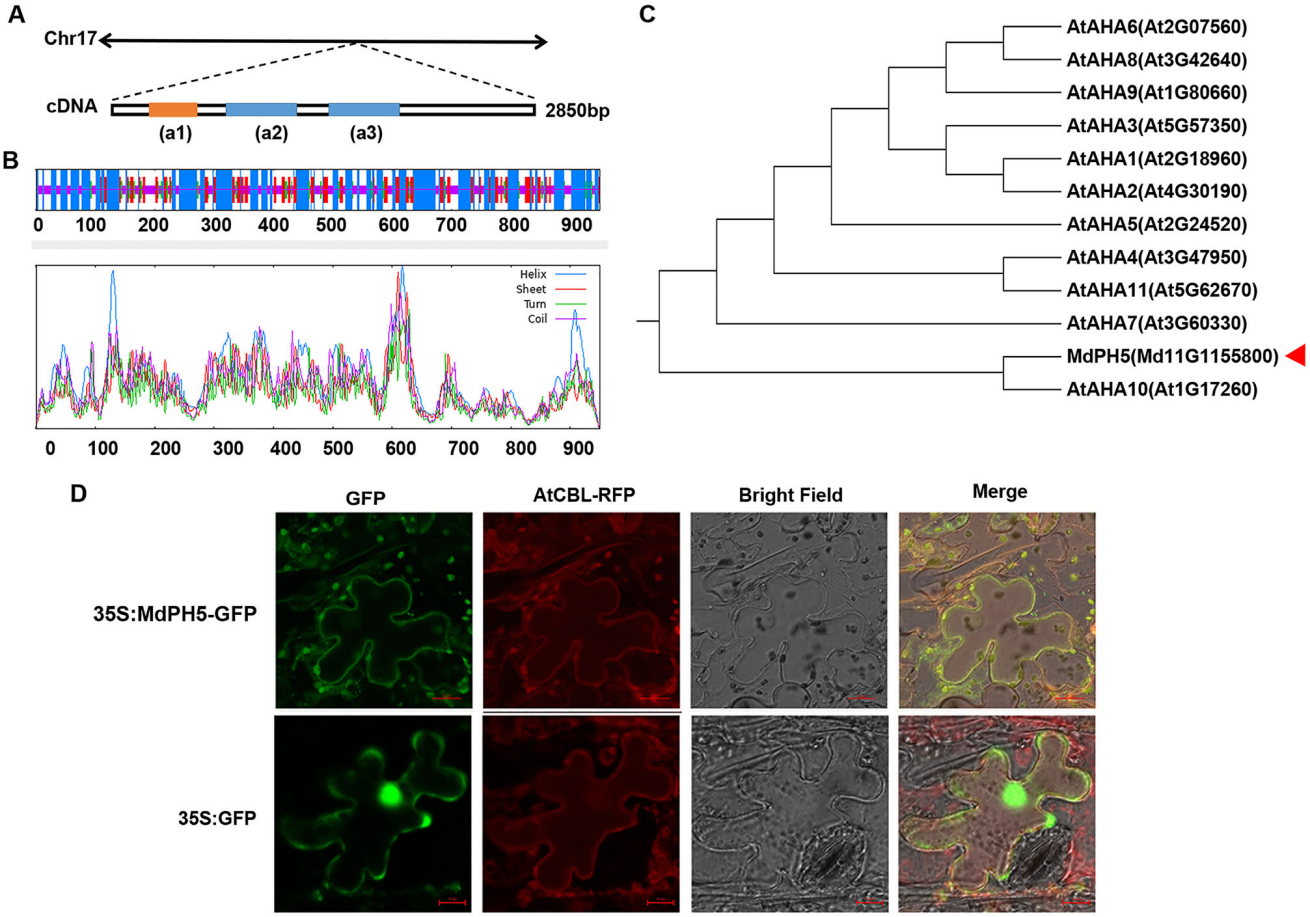
Bioinformatics analysis of the *MdPH5* gene and subcellular localization of the *MdPH5* protein. **A** Conserved sequence of the *MdPH5* gene; a1 refers to ATPase-N, a2 refers to E1-E2_ ATPase, and a3 refers to hydrolase. **B** Predicted secondary structures of the *MdPH5* protein. The numbers denote the length of amino acids. **C** Phylogenetic tree of the *MdPH5* protein and ATPase of the *Arabidopsis thaliana* P_3A_ subfamily. **D** Subcellular localization of the *MdPH5* protein with a AtCBL tonoplast marker. Herein, lines and boxes highlight the position of vacuoles exhibiting green and red fluorescence, respectively. 35S::GFP refers to the control group, scale bar = 10 µm, and B represents a bright field

The cDNA of *MdPH5* was 2850 bp in full length and encodes for 950 amino acids. As shown in Fig. 2A, the cDNA of *MdPH5* comprised three conserved domains. Herein, the secondary protein structure of MdPH5 was predicted. The results showed that random coils (69.88%), alpha-helices (30.00%), extended-strands (17.05%), and beta-turns (5.58%) were dominant in the secondary protein structure of MdPH5 (Fig. 2B).

A phylogenetic tree was established, using MEGA-X, to investigate the genetic correlation of MdPH5 with the P_3A_ subfamily ATPases in *Arabidopsis thaliana*. These results indicated that MdPH5 (MD17G1155800) had the closest genetic correlation with At1G17260 (Fig. 2C).

As *MdPH5* encodes a P-ATPase, *pCaMV35S::MdPH5-GFP* fusion vectors were developed and visualized by transient expression in leaves of *N. benthamiana*, with *pCaMV35S::GFP* as a negative control, so as to determine the subcellular localization of MdPH5. Herein, different profiles of the vacuolar membrane-labeled red fluorescence and the green fluorescence of the MdPH5-GFP protein were observed. Additionally, the fluorescence signal of MdPH5-GFP overlapped with that of the AtCBL-labeled vacuolar membrane marker (Fig. 2D). Therefore, it could be concluded that MdPH5 is localized to the vacuolar membrane.

### Role of *MdPH5* in regulating malate accumulation and vacuolar pH in apple

**Fig. 3:**
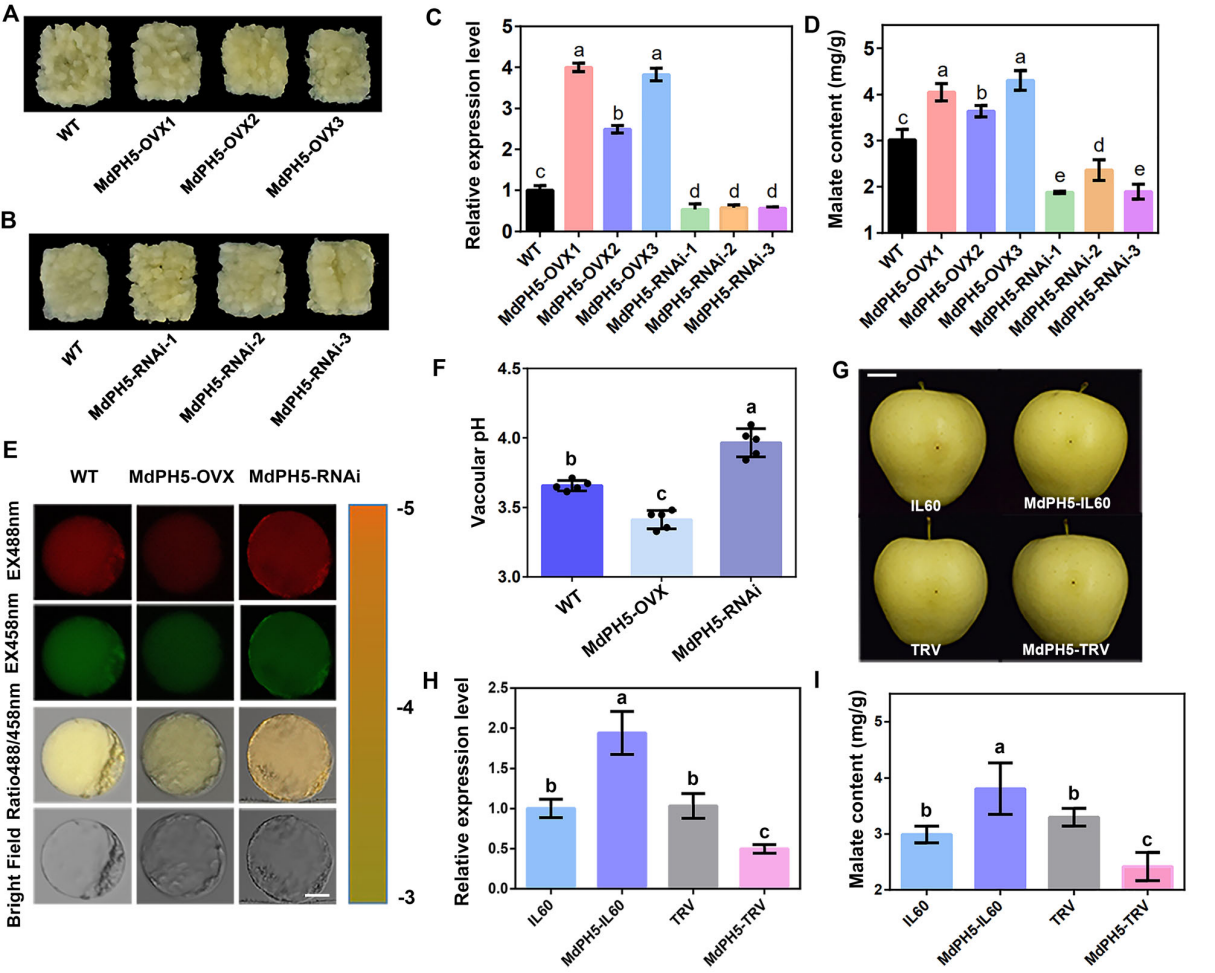
Functions of *MdPH5*. **A**, **B** Transgenic callus materials obtained. **C** The gene expression of *MdPH5* in the wild-type (WT) and *MdPH5* transgenic apple calli (overexpression and antisense) determined by RT-qPCR. **D** Malate contents in MdPH5-OVX and MdPH5-RNAi transgenic apple calli. **E** Emission intensities of protoplast vacuoles in WT and transgenic calli MdPH5-OVX and MdPH5-RNAi loaded with wit20,70-bis-(2-carbox-yethyl)-5-(6)-carboxyfluorescein at 488 nm (first column) and 458 nm (second column). The pseudo-color scale on the right indicates the fluorescence intensity. Scale bar = 10 µm. Lowercase letters indicate significant differences at *P* < 0.05. All values are the mean ± SD of three independent replicates. **F** Quantification of the luminal pH in vacuoles of WT and transgenic calli MdPH5-OVX and MdPH5-RNAi. Error bars represent the SE of 5 measurements from 30 individual intact vacuoles. Lowercase letters indicate significant differences at *P* < 0.05. **G** Apple fruit injected with plasmid mixtures (IL60: IL60-1 + IL60-2; MdPH5-IL60: IL60-1 + MdPH5-IL60-2). An empty IL60 vector was used as the control group. A solution containing agrobacterium cells (TRV: TRV1 + TRV2; MdPH5-TRV: TRV1 + MdPH5-TRV2) was injected into the apple tissue, with an empty TRV vector serving as the control group. Scale bar = 2 cm. Measured expression levels of *MdPH5* (**H**) and malate contents (**I**) at the injection sites. Significant differences are indicated by the use of lowercase letters if *P* < 0.05. All values are the mean ± SD (n = 5)

Transgenic calli with overexpression or silencing of *MdPH5* were acquired to investigate the functions of the gene (Fig. 3A, B). The expression of *MdPH5* in the overexpression or silencing groups was significantly higher and lower than that in the control group, respectively, indicating successful preparation of the gene overexpression or silencing materials (Fig. 3C). Compared with the control group, the overexpression or silencing groups exhibited increased and decreased malate contents (Fig. 3D), respectively, indicating that *MdPH5* favors malate accumulation in calli.

The effects of *MdPH5* on vacuolar pH were investigated on the basis of BCECF [2′,7′-Bis(2-carboxyethyl)-5(6)-carboxyfluorescein], which is a ratiometric fluorescent pH indicator. The average vacuolar pH value of WT apple calli was 3.66, whereas that of *MdPH5* overexpression or silencing apple calli was 3.96 and 3.41, respectively (Fig. 3E, F). Overall, it could be inferred that *MdPH5* regulates malate accumulation and vacuolar pH in apple calli.

To further verify the effects of *MdPH5* on malate, the expression of *MdPH5* in apple was tuned by a virus-vector-based transformation method. Two virus constructors (MdPH5-IL60 and MdPH5-TRV) were injected into the fruit by agrobacterium transformation, with empty vectors as the control (Fig. 3G). The expression level of *MdPH5* was aligned with expectation by a 7-day incubation in darkness (Fig. 3H). Then the malate content in the MdPH5-IL60 group was measured and compared with that in the control group. As indicated, the average malate content in the MdPH5-IL60 group increased by 0.671 mg/g. Similarly, the average malate content in the MdPH5-TRV group decreased by 0.670 mg/g, which was consistent with that in the transgenic calli (Fig. 3I).

### Role of MdMYB73 in *MdPH5* expression

Previous studies have shown that some TFs can directly activate the expression of several vacuolar proton pump subunit genes (Hu et al*.* 2016, 2017). To elucidate the transcriptional regulatory mechanism of *MdPH5* in apple, *cis*-acting element analysis of its promoter was conducted and the results demonstrated the presence of abundant MYB-binding *cis* elements in the *MdPH5* promoter. This may be attributed to the presence of some MYB TFs that are located upstream of *MdPH5* and regulate its expression. After that, the MYB TFs interacting with *MdPH5* were identified by yeast one hybrid assays. These results demonstrated that MdMYB73, which was previously identified as a major TF regulating malate, is indeed a candidate partner.

**Fig. 4:**
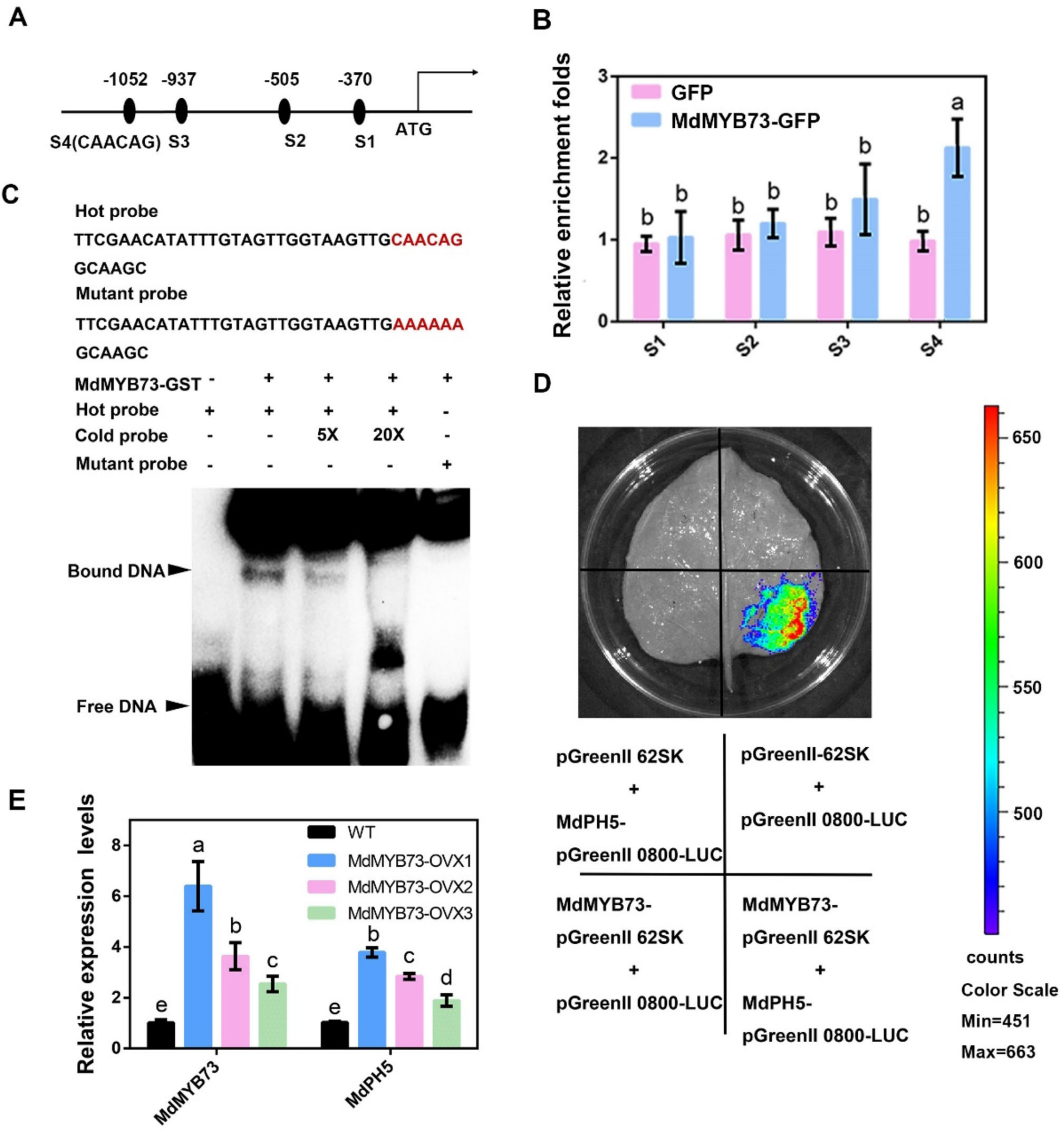
MdMYB73 regulates malate accumulation by binding to *MdPH5*. **A** The putative MYB-binding element on the *MdPH5* promoters; the numbers represent the integrated position. **B** ChIP-qPCR results indicating the enrichments of the target gene promoters in the *35S::MdMYB73-GFP* transgenic apple seedlings compared with the *35S::GFP* transgenic apple seedlings. **C** Results of electrophoretic mobility shift assays of the interaction between MdMYB73 and labeled DNA probes in the *MdPH5* promoters. Lane 1 of each blot shows the labeled DNA probes without the MdMYB73 protein; lane 2 shows the labeled DNA probes and the MdMYB73 protein without a competitor. Different amounts (× 5 and × 10) of unlabeled DNA fragments were added as cold competitors. **D** Results of LUC activity assays indicating that MdMYB73 enhances the basal activity of the *MdPH5* promoters. **E** The expression level of *MdPH5* in MdMYB73 transgenic apple seedlings. A significant difference is indicated by distinct lowercase letters at *P* < 0.05. All values are the mean ± SD (n = 3)

Chromatin immunoprecipitation (ChIP)-PCR assays were employed to investigate the in vivo binding of MdMYB73 to the *MdPH5* promoter. Herein, *35S::MdMYB73-GFP* and 35S::GFP transgenic apple cultures were used. The MYB-binding site element (CAACAG) in Region S4 of the *MdPH5* promoter was enriched in the *35S::MdMYB73-GFP* transgenic cultures, though the MYB-binding element in other three regions was not enriched (Fig. 4A, B). The results provided in vivo evidence for the binding of MdMYB73 to the *MdPH5* promoter.

An electrophoretic mobility shift assay (EMSA) was performed to investigate the in vitro binding of MdMYB73 to the *MdPH5* promoter. As observed, MdMYB73 bound to the *MdPH5* promoter fragments containing the CAACAG motifs, and the level of a specific DNA-MdMYB73 protein complex decreased with the increase in the number of unlabeled MYB-competing probes with the same sequence. However, these complexes were not observed if the CAACAG motif was changed to the AAAAAA motif (Fig. 4C). These results provided in vitro evidence for the binding of MdMYB73 to the *MdPH5* promoter.

The luciferase (LUC) transactivation assay was employed to clarify the effects of MdMYB73 on the activity of the *MdPH5* promoter. As indicated, promoter-LUC reporter plasmids were expressed, transiently, in the leaves of *N. benthamiana,* via transfection with *Agrobacterium tumefaciens* strain GV3101. The inflitrated leaves containing the sequences of *MdPH5* promoter showed higher LUC activity (Fig. 4D). In summary, these findings support the notion that *MdPH5* transcription is activated by MdMYB73.

In addition, the expression levels of *MdMYB73* and *MdPH5* in *35S::MdMYB73-GFP* transgenic apple plantlets were investigated. As indicated, the expression levels of both genes in the transgenic apple plantlets were significantly higher than those in the control group (Fig. 4E).

### Role of MdMYB73 as an upstream gene of *MdPH5* in regulating malate accumulation

**Fig. 5:**
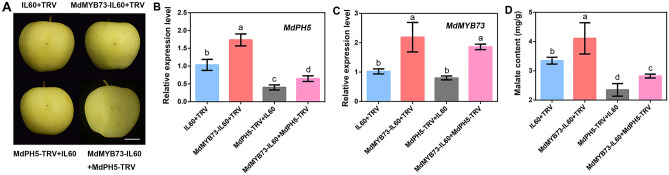
Verification of the upstream and downstream relationship of *MdMYB73* and *MdPH5* genes. **A** Injected apples kept in darkness for a week. Scale bar = 2 cm. **B**, **C** Expression of related genes in apple fruit after injection. **D** Malate contents in different groups. Error bars represent the SE of five measurements from at least independent biological replicates

To clarify the regulatory role of *MdMYB73* and *MdPH5* in malate accumulation, four virus builders (IL60 + TRV, MdPH5-TRV, MdMYB73-IL60, and MdPH5-TRV + MdMYB73-IL60) were used for fruit injection tests (Fig. 5A). The results of RT-qPCR showed that, compared with IL60 + TRV, the relative transcription levels of *MdMYB73* and *MdPH5* were consistent with expectations (Fig. 5B, C) and the transcription level of *MdPH5* decreased with downregulated expression of *MdMYB73*. Subsequently, the malate content was determined. As indicated, the malate concentration in the injection area of MdMYB73-IL60 was higher than that in the control group, whereas that in the injection area of MdPH5-TRV was lower than that in the injection area of the control group. Additionally, the malate content in the injection area of MdMYB73-IL60 + MdPH5-TRV was higher than that in the injection area of MdPH5-TRV, but slightly lower than that in the injection area of the control group. In other words, MdPH5-TRV may partially offset the effects of MdMYB73-IL60 in the injection area of MdMYB73-IL60 + MdPH5-TRV (Fig. 5D). In summary, MdMYB73 is genetically upstream of *MdPH5* in the regulation of malate accumulation in apple fruit.

## Discussion

Acidity is one of the most important quality-related features of apple, which directly affects fruit flavor and quality. Apple contains abundant organic acids, among which malate is the dominant species, as it accounts for more than 90% of the total organic acids (Yu et al. 2021). Widely distributed in various plant tissues and organs, malate has various important functions in the life cycle of plants (Fernie et al. 2004; Noguchi and Yoshida 2008; Sweetman et al. 2009; Bai et al. 2015; Hu et al*.* 2017; Dong et al. 2018). Vacuolar proton pumps and malate transporters are essential key factors influencing the transport of malate into vacuoles (Terrier et al. 2001; Bai et al. 2015; Ma et al. 2015, 2019; Hu et al. 2016). In this study, MdPH5, a P-type ATPase, was shown to promote vacuolar acidification and malate accumulation in apple calli and fruit. As an upstream MYB TF of *MdPH5*, MdMYB73 binds to the *MdPH5* promoter and activates its expression, thereby facilitating malate accumulation. In addition, the expression of *MdPH5* was positively correlated with malate content, at both the developmental and post-harvest stages.

Vacuolar proton pumps have significant effects on fruit acidity, as they can deliver H^+^ to acidifying vacuoles, thereby facilitating the accumulation of organic acids (Emmerlich et al. 2003). As MdPH5 acidifies apple vacuoles, its homolog PhPH5 acidifies petal cell vacuoles. Nevertheless, *PhPH5* affects petal color rather than the accumulation of organic acids (Faraco et al. 2014). P-type ATPases promote the accumulation of organic acids and play an important role in fruits. *Ma10*, a candidate gene for the acidity of apple fruit, encodes a P_3A_-type ATPase proton pump that promotes the transport of malate into vacuoles and vacuole acidification (Ma et al. 2019). In pears, the *PH5* gene also promotes malate accumulation (Song et al. 2021). Overall, these genes exhibit similar functions to MdPH5 in apple. However, downregulated expression of *CitPH5* led to the formation of some low-acid varieties of some fruits, including lemon, orange, pummelo, and rangpur lime (Strazzer et al. 2019). Despite that citric acid is the dominant organic acid in these fruits, this study focused on the effects of MdPH5 on malate accumulation, but not citric acid accumulation in apple. In addition, the *cis* element of *MdPH5* promoter was further analyzed using the PlantCARE. As shown in Supplemental Table 2, there are many *cis* elements involved in light responsiveness, drought inducibility, and hormone responsiveness (jasmonic acid, salicylic acid, and auxin). These findings further suggest that the MdPH5-mediated malate metabolism may be induced by various signals, such as plant hormones, stress, and light.

**Fig. 6:**
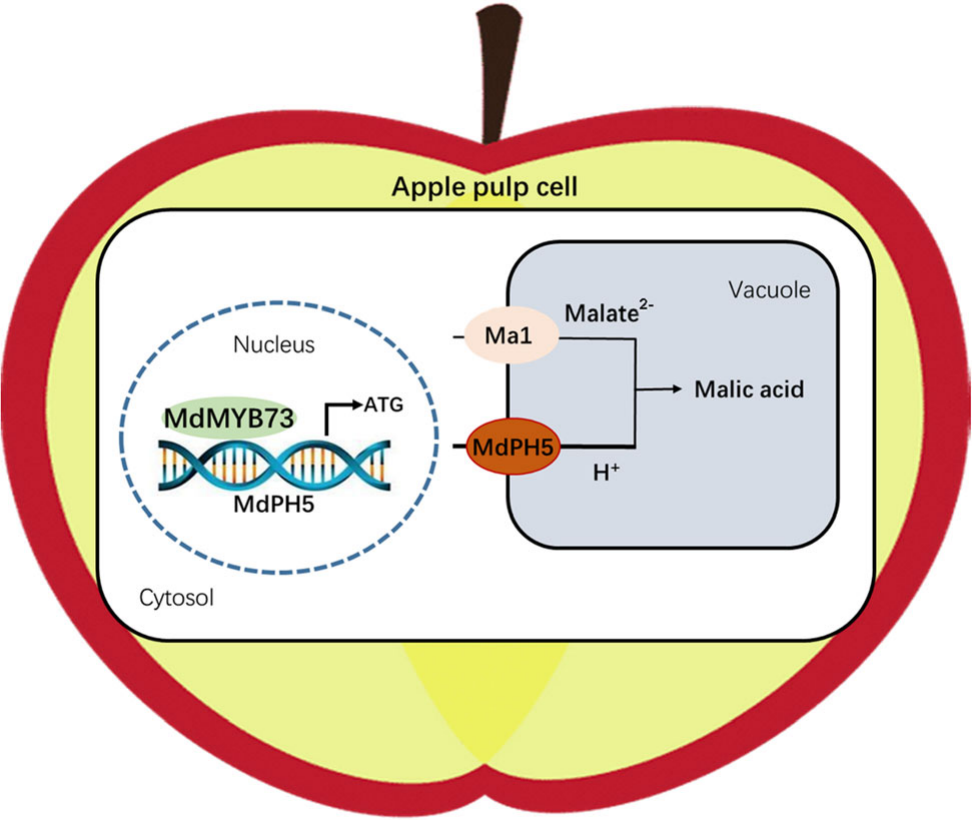
Working model showing that MdMYB73 binds to the *MdPH5* promoter to regulate malate accumulation in apple

Malate content is subjected to the synergistic effects of environmental factors, developmental and metabolic signaling pathways, and corresponding TFs. TFs can regulate malate accumulation either individually or in the form of complexes (Etienne et al. 2013; Hu et al. 2016, 2017; Li et al. 2020). In apple, the transcription activities of malate transporters and proton pumps can be significantly tuned by regulating MdMYB1, MdMYB44, and MdMYB73, which can effectively regulate malate accumulation and vacuolar acidification (Hu et al. 2016, 2017; Jia et al. 2021). However, MdMYB1 and MdMYB73 are positive regulators, whereas MdMYB44 is a negative regulator, and they have different downstream target genes. Indeed, the direct downstream target genes of MdMYB1 are *MdVHA-B1*, *MdVHA-E*, *MdVHF1,* and *MdtDT*, whereas MdMYB44 inhibits the promoter activity of *MdVHA-A3*, *MdVHA-D2*, *Ma10*, and *MdALMT9*. In contrast, MdMYB73 directly activates the expression of *MdVHA-A*, *MdVHP1,* and *MdALMT9*. In this study, *MdPH5* was identified to be another downstream target gene of MdMYB73. Similarly, MdMYB73 could regulate MdPH5 to promote malate accumulation (Hu et al. 2016, 2017; Jia et al. 2021; Fig. 6). Notably, MdMYB44 has been demonstrated to regulate both V-ATPase and P-ATPase genes (Jia et al. 2021). To date, only a few downstream V-ATPase target genes for MdMYB73 TF have been reported. MdPH5 is a P_3A_-type ATPase, suggesting that MdMYB73 can also simultaneously regulate both types of proton pumps.

Besides MYB TFs, WRKY, bHLH, ERF, and other TFs are also involved in the regulation of organic acid accumulation and vacuole acidification. An InDel in the *SlALMT9* promoter disrupts a W-box binding site, thereby preventing the binding of the WRKY TFs (Ye et al. 2017). In apple, MdbHLH3 (bHLH TF) directly regulates the expression of *MdcyMDH*, which is a cytosolic malate dehydrogenase gene, to mediate carbohydrate allocation and malate accumulation (Yu et al. 2021). CitERF13 (citrus TF) regulates citrate accumulation by directly activating *CitVHA-c4*, which is a vacuolar proton pump gene (Li et al. 2016). Also, the formation of MBW complexes can directly regulate the expression of downstream acid-related target genes. As reported, MdbHLH3, MdbHLH49, and MdCIbHLH1 interact with MdMYB1, MdMYB44, and MdMYB73, respectively, and enhance the activities of corresponding MYB TFs to further regulate the activities of downstream genes, including malate transporters and vacuolar proton pumps (Xie et al. 2012; Hu et al. 2016, 2017; Jia et al. 2021). Therefore, there may be more complex regulatory relationships in addition to the proposed MdMYB73-MdPH5 malate regulatory pathway. In addition, the *cis* element of *MdMYB73* promoter was further analyzed using the PlantCARE. As shown in Supplemental Table 3, there are many *cis* elements involved in light responsiveness and drought inducibility. These results further suggest that the proposed MdMYB73-MdPH5 malate regulatory pathway may be induced by light and drought signals.

Fruit quality is one of the most concerned features in fruit breeding. It has been demonstrated that organic acids significantly affect fruit flavor. Malate is one of the most important organic acids in apple, while the genetic basis for its accumulation in apple vacuoles remains unclear (Wu et al. 2007; Zhang et al. 2012). In this study, MdPH5, a P_3A_-type vacuolar proton pump, was demonstrated to promote malate accumulation and vacuolar acidification, and MdMYB73, an upstream MYB TF of *MdPH5*, can facilitate malate accumulation by binding to its promoter and activating its expression (Fig. 6). This study provides a new method for acidity regulation of apple fruit, and may facilitate the development of new varieties with improved flavor and stress resistance.

### Supplementary Information

Below is the link to the electronic supplementary material.

Supplementary file1 (DOCX 5613 KB)

## Data Availability

The data that support the findings of this study are available from the corresponding author upon reasonable request.

## References

[CR1] Amato A, Cavallini E, Walker AR, Pezzotti M, Bliek M, Quattrocchio F (2019). The MYB5-driven MBW complex recruits a WRKY factor to enhance the expression of targets involved in vacuolar hyper-acidification and trafficking in grapevine. Plant J.

[CR2] Bai Y, Dougherty L, Cheng L, Zhong GY, Xu K (2015). Uncovering co-expression gene network modules regulating fruit acidity in diverse apples. BMC Genom.

[CR3] Cavallini E, Zenoni S, Finezzo L, Guzzo F, Zamboni A, Avesani L (2014). Functional diversification of grapevine MYB5a and MYB5b in the control of flavonoid biosynthesis in a petunia anthocyanin regulatory mutant. Plant Cell Physiol.

[CR4] Cohen S, Itkin M, Yeselson Y, Tzuri G, Portnoy V, Harel-Baja R (2014). The PH gene determines fruit acidity and contributes to the evolution of sweet melons. Nat Commun.

[CR5] Dong H, Bai L, Zhang Y, Zhang G, Mao Y, Min L, Xiang FY, Qian DD, Zhu XH, Song CL (2018). Modulation of guard cell turgor and drought tolerance by a peroxisomal acetate-malate shunt. Mol Plant.

[CR6] Emmerlich V, Linka N, Reinhold T, Ekkehard Neuhaus H (2003). The plant homolog to the human sodium/dicarboxylic cotransporter is the vacuolar malate carrier. Proc Natl Acad Sci USA.

[CR7] Etienne A, Genard M, Lobit P, Mbeguie AMD, Bugaud C (2013). What controls fleshy fruit acidity? A review of malate and citrate accumulation in fruit cells. J Exp Bot.

[CR8] Faraco M, Spelt C, Bliek M, Verweij W, Hoshino A, Espen L, Prinsi B, Jaarsma R, Tarhan E, Sansebastiano GPD (2014). Hyperacidification of vacuoles by the combined action of two different P-ATPases in the tonoplast determines flower color. Cell Rep.

[CR9] Fernie AR, Carrari F, Sweetlove LJ (2004). Respiratory metabolism: glycolysis, the TCA cycle and mitochondrial electron transport. Curr Opin Plant Biol.

[CR10] Hu DG, Sun CH, Ma QJ, You CX, Cheng L, Hao YJ (2016). MdMYB1 regulates anthocyanin and malate accumulation by directly facilitating their transport into vacuoles in apples. Plant Physiol.

[CR11] Hu DG, Li YY, Zhang QY, Li M, Sun CH, Yu JQ, Hao YJ (2017). The R2R3-MYB transcription factor MdMYB73 is involved in malate accumulation and vacuolar acidification in apple. Plant J.

[CR12] Huang XY, Wang CK, Zhao YW, Sun CH, Hu DG (2021). Mechanisms and regulation of organic acid accumulation in plant vacuoles. Hortic Res.

[CR13] Hurth MA, Suh SJ, Kretzschmar T, Geis T, Bregante M, Gambale F, Martinoia E, Ekkehard Neuhaus H (2005). Impaired pH homeostasis in Arabidopsis lacking the vacuolar dicarboxylate transporter and analysis of carboxylic acid transport across the tonoplast. Plant Physiol.

[CR14] Jia D, Wu P, Shen F, Li W, Zheng X, Wang Y, Yuan YB, Zhang XZ, Han ZH (2021). Genetic variation in the promoter of an R2R3-MYB transcription factor determines fruit malate content in apple (*Malus domestica* Borkh.). Plant Physiol.

[CR15] Kasajima I, Sasaki K (2016). A chimeric repressor of petunia PH4 R2R3-MYB family transcription factor generates margined flowers in torenia. Plant Signal Behav.

[CR16] Kovermann P, Meyer S, Hortensteiner S, Picco C, Scholz-Starke J, Ravera S, Lee Y, Martinoia E (2007). The Arabidopsis vacuolar malate channel is a member of the ALMT family. Plant J.

[CR17] Li SJ, Liu XJ, Xie XL, Sun CD, Grierson D, Yin XR, Chen KS (2015). CrMYB73, a PH -like gene, contributes to citric acid accumulation in citrus fruit. Sci Hortic.

[CR18] Li SJ, Yin XR, Xie XL, Allan AC, Ge H, Shen SL (2016). The Citrus transcription factor, CitERF13, regulates citric acid accumulation via a protein-protein interaction with the vacuolar proton pump, CitVHA-c4. Sci Rep.

[CR19] Li CL, Dougherty L, Coluccio AE, Meng D, El-Sharkawy I, Borejsza-Wysocka E, Liang D, Piñeros MA, Xu KN, Cheng LL (2020). Apple ALMT9 requires a conserved C-terminal domain for malate transport underlying fruit acidity. Plant Physiol.

[CR20] Ma B, Liao L, Zheng H, Chen J, Wu B, Ogutu C, Li SH, Korban SS, Han YP (2015). Genes encoding aluminum-activated malate transporter II and their association with fruit acidity in apple. Plant Genome.

[CR22] Ma B, Liao L, Fang T, Peng Q, Ogutu C, Zhou H, Ma FW, Han YP (2019). A * Ma10 * gene encoding P-type ATPase is involved in fruit organic acid accumulation in apple. Plant Biotechnol J.

[CR23] Martinoia E (2018). Vacuolar transporters—companions on a longtime journey. Plant Physiol.

[CR24] Martinoia E, Maeshima M, Neuhaus HE (2007). Vacuolar transporters and their essential role in plant metabolism. J Exp Bot.

[CR25] Meyer S, Scholz-Starke J, De Angeli A, Kovermann P, Burla B, Gambale F, Martinoia E (2011). Malate transport by the vacuolar AtALMT6 channel in guard cells is subject to multiple regulation. Plant J.

[CR26] Noguchi K, Yoshida K (2008). Interaction between photosynthesis and respiration in illuminated leaves. Mitochondrion.

[CR27] Pedersen CN, Axelsen KB, Harper JF, Palmgren MG (2012). Evolution of plant p-type ATPases. Front Plant Sci.

[CR28] Song XJ, Chen YC, Lu ZH, Zhao GP, Wang XL, Zhai R, Wang ZG, Yang CQ, Xu LF (2022). PbPH5, an H^+^ P-ATPase on the tonoplast, is related to malic acid accumulation in pear fruit. J Integr AGR.

[CR29] Strazzer P, Spelt CE, Li S, Bliek M, Federici CT, Roose ML, Koes R, Quattrocchio FM (2019). Hyperacidification of citrus fruits by a vacuolar proton-pumping P-ATPase complex. Nat Commun.

[CR30] Sundaramoorthy J, Park GT, Lee GD, Kim GH, Seo SH, Song JT (2020). A P_3A_-type ATPase and an R2R3-MYB transcription factor are involved in vacuolar acidification and flower coloration in soybean. Front Plant Sci.

[CR31] Sweetman C, Deluc LG, Cramer GR, Ford CM, Soole KL (2009). Regulation of malate metabolism in grape berry and other developing fruits. Phytochemistry.

[CR32] Terrier N, Sauvage FX, Ageorges A (2001). Changes in acidity and in proton transport at the tonoplast of grape berries during development. Planta.

[CR33] Verweij W, Spelt C, Di Sansebastiano GP, Vermeer J, Reale L, Ferranti F, Koes R, Quattrocchio F (2008). An H^+^ P-ATPase on the tonoplast determines vacuolar pH and flower colour. Nat Cell Biol.

[CR34] Wu J, Gao H, Zhao L, Liao X, Chen F, Wang Z, Hu X (2007). Chemical compositional characterization of some apple cultivars. Food Chem.

[CR35] Xie XB, Li S, Zhang RF, Zhao J, Chen YC, Zhao Q, Yao YX, You CX, Zhang XS, Hao YJ (2012). The bHLH transcription factor MdbHLH3 promotes anthocyanin accumulation and fruit colouration in response to low temperature in apples. Plant Cell Environ.

[CR36] Ye J, Wang X, Hu T, Zhang F, Wang B, Li C, Yang TX, Li HX, Lu YG, Giovannoni JJ, Zhang YY, Ye ZB (2017). An inDel in the promoter of al-ACTIVATED MALATE TRANSPORTER9 selected during tomato domestication determines fruit malate contents and aluminum tolerance. Plant Cell.

[CR37] Yu JQ, Gu KD, Sun CH, Zhang QY, Wang JH, Ma FF, You CX, Hu DG, Hao YJ (2021). The apple bHLH transcription factor MdbHLH3 functions in determining the fruit carbohydrates and malate. Plant Biotechnol J.

[CR38] Zhang Q, Li J, Zhao Y, Korban SS, Han Y (2012). Evaluation of genetic diversity in Chinese wild apple species along with apple cultivars using SSR markers. Plant Mol Biol Rep.

