# Supplementary material for: Regulation of a vacuolar proton-pumping P-ATPase MdPH5 by MdMYB73 and its role in malate accumulation and vacuolar acidification
Source: aBIOTECH. 2023 Sep 22;4(4):303–14. doi: 10.1007/s42994-023-00115-7 (PMC10721769; doi:10.1007/s42994-023-00115-7)
Supplement: Supplementary file 1 — Supplementary file1 (DOCX 5613 KB) [file 42994_2023_115_MOESM1_ESM.docx]

**Abiotech Supplemental Information Figure S1-S4 and Table S1-S3**

**The Title**

Regulation of a vacuolar proton-pumping P-ATPase MdPH5 by MdMYB73 and its role in malate accumulation and vacuolar acidification

**The full names of all the authors**

Xiao-Yu Huang^#^, Ying Xiang^#^, Chu-Kun Wang, Jia-Hui Wang, Wen-Yan Wang, Xiao-Long Liu, Quan Sun^*^, Da-Gang Hu^*^

**The names and address of the institution**

National Research Center for Apple Engineering and Technology, Shandong Collaborative Innovation Center of Fruit & Vegetable Quality and Efficient Production, College of Horticulture Science and Engineering, Shandong Agricultural University, Tai’an, Shandong 271018, China

**Corresponding authors**

Name: Da-Gang Hu (fap_296566@163.com), Quan Sun (sunquan@sdau.edu.cn)

Tel: +86-538-824-6151

Fax number: +86-538-824-2364

Address: National Research Center for Apple Engineering and Technology; College of Horticulture Science and Engineering, Shandong Agricultural University, Tai’an, Shandong 271018, China

**
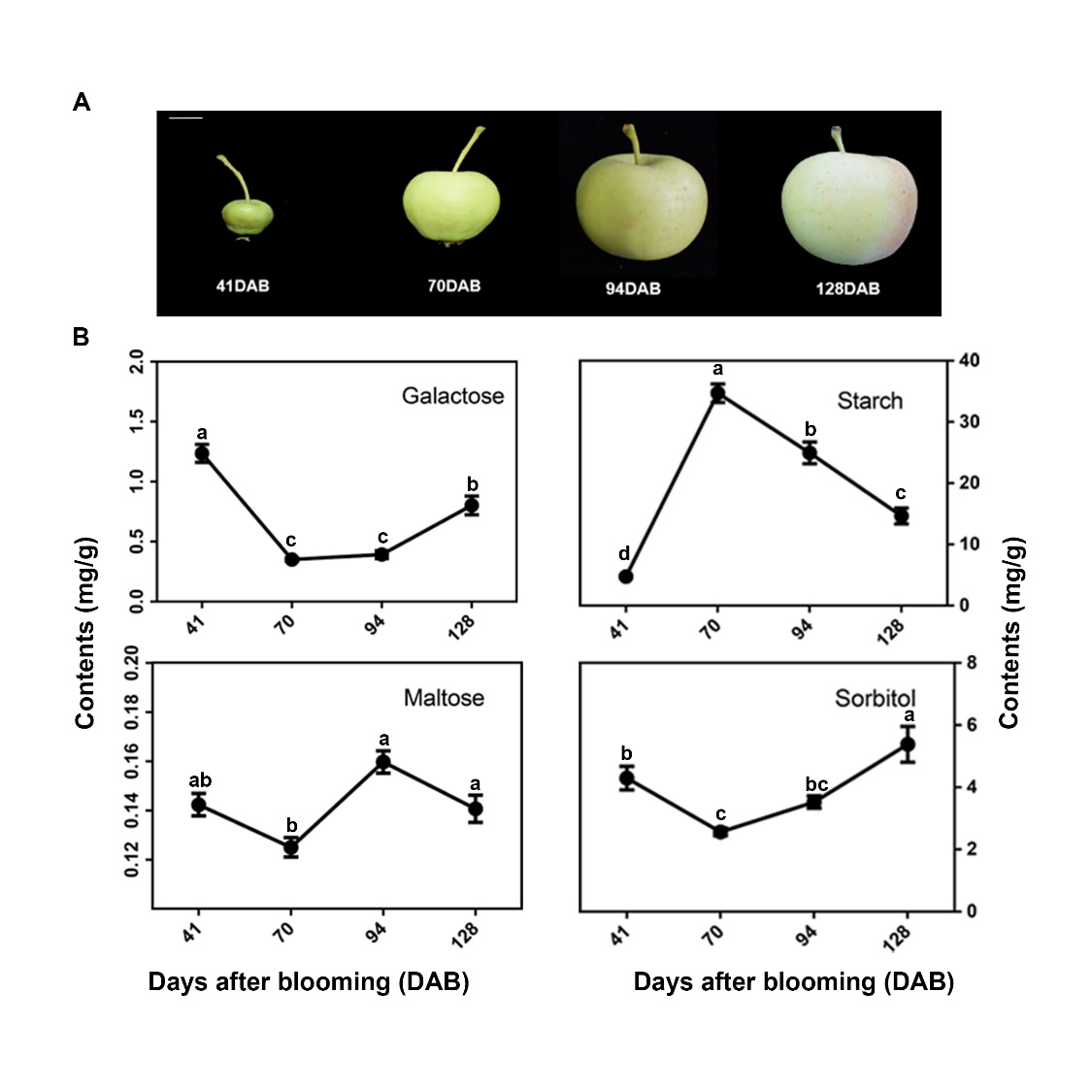
**

**Fig S1.** Metabolites contents in apple fruit on different DAB. **A** Apple fruit at different developmental stages. Scale bar = 2 cm. **B** Contents of various metabolites in apple fruit on different DAB.


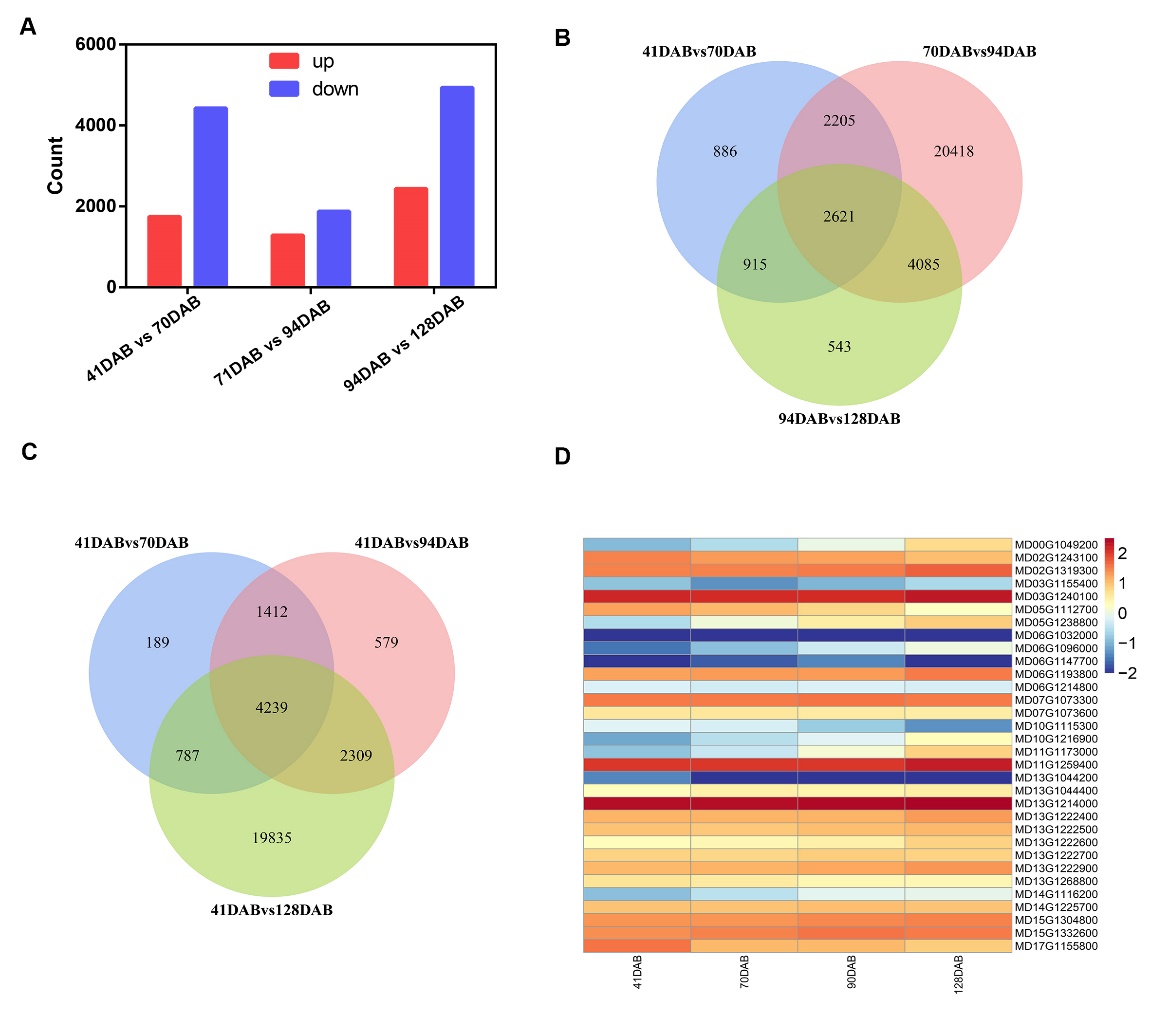


**Fig S2.** Transcriptome analysis of apple fruit on different DAB. **A** Number of differential genes in apple fruits on different DAB. **B, C** Malate contents in apple fruit on different DAB. **D** Heatmaps of 32 genes possibly associated with malate at different developmental stages.


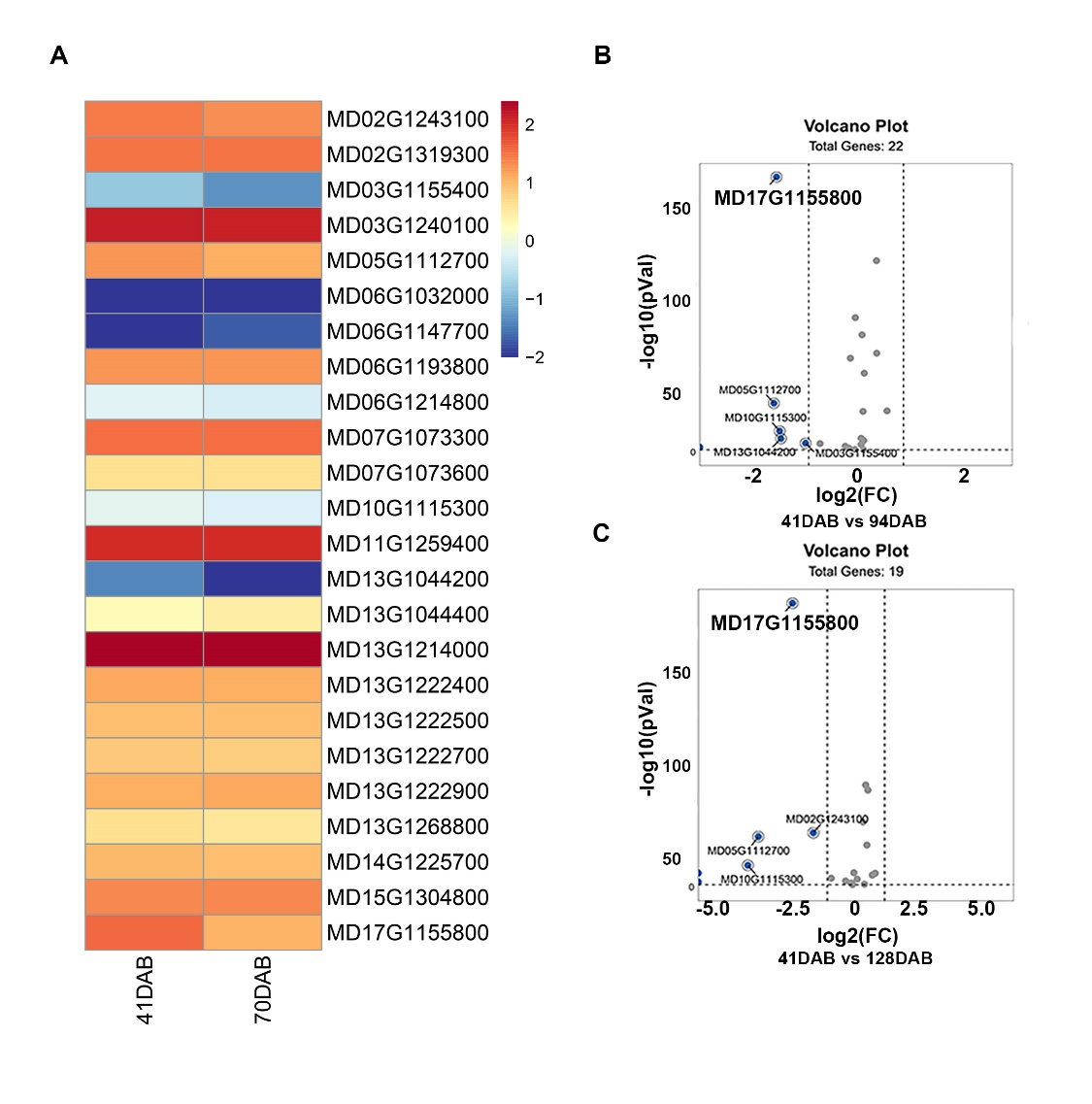


**Fig S3.** Heat maps (**A**) and volcano maps (**B**) related to malate genes in 40 DAB and 70 DAB.


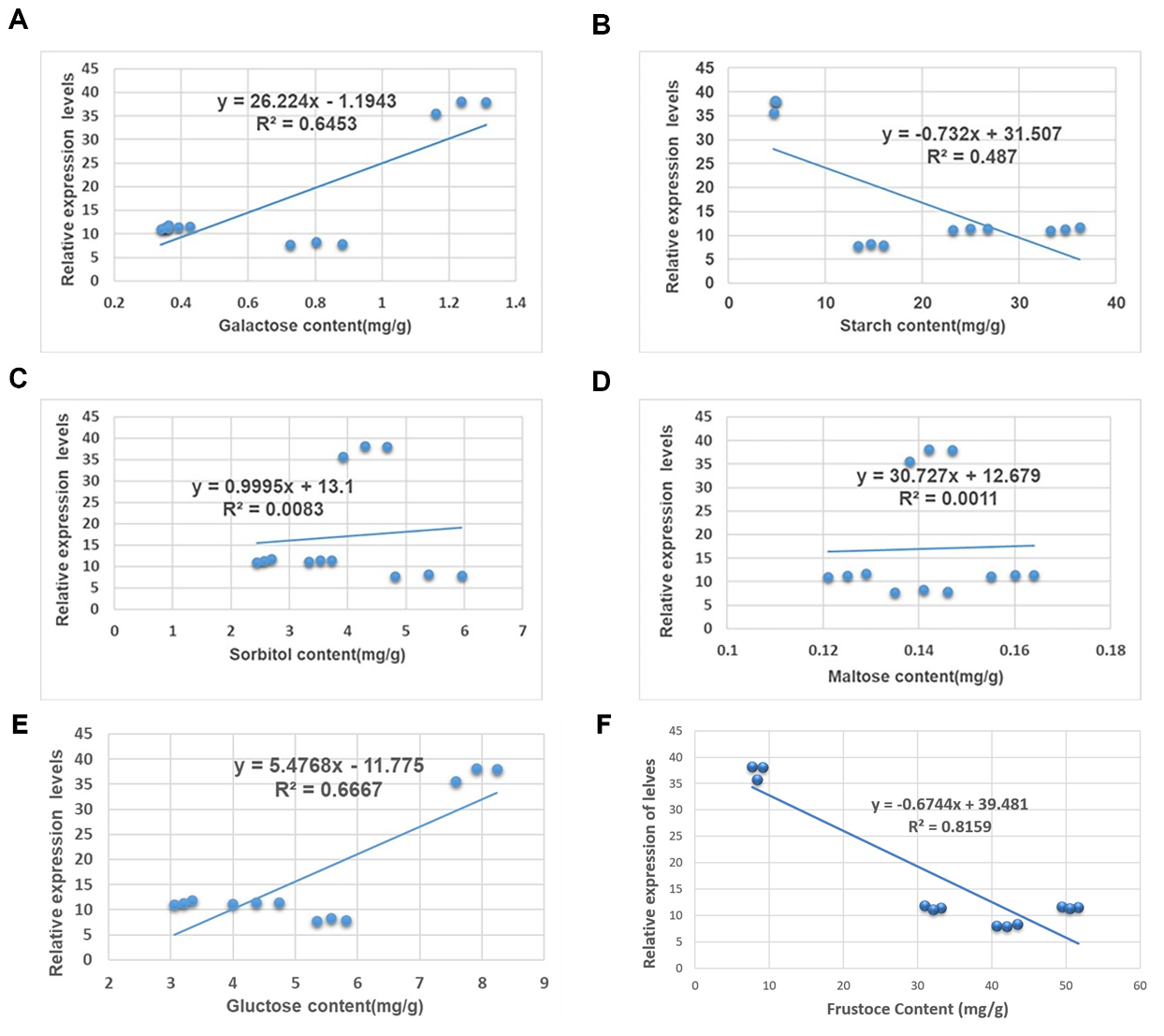


**Fig S4.** Correlation of the expression level of *MdPH5* with different carbohydrate substances at different developmental stages.

Correlation of the expression level of *MdPH5* with galactose **A**, starch **B**, sorbitol **C**, maltose **D**, gluctose **E**, and frustoce **F** at different developmental stages.

**Supplemental Table 1.** Primers used for gene expression analysis and vector construction in this study.

| **Prime name** | **Prime sequence** |
| --- | --- |
| MdPH5-F | TGCCCTCCTAAAATAAGTGAACTT |
| MdPH5-R | GATTTAAGCTTTCAAATCCTG |
| MdMYB73-F | AGTAGAGAGCAGATATCCGAC |
| MdMYB73-R | ATCATGATCATGCACTGCTAC |
| ProMdPH5-F | TGCCCTCCTAAAATAAGTGAACTT |
| ProMdPH5-R | GATTTAAGCTTTCAAATCCTG |
| MdMYB73-F(RT-qPCR) | AGTAGAGAGCAGATATCCGAC |
| MdMYB73-R(RT-qPCR) | ATCATGATCATGCACTGCTAC |
| MdPH5-F(RT-qPCR) | ATAGCGTCTCCTTCCTTACT |
| MdPH5-R(RT-qPCR) | AGTGAGCACTTGGGGAAGA |
| MdPH5-F(LUC) | GTCGACTTTATTCGAACATATTTGTAG |
| MdPH5-R(LUC) | CCCGGGGATTTAAGCTTTCAAAT |
| MdPH5-F(EMSA) | CAAACATTTTTGTATATTTCTTTTTATT |
| MdPH5-R(EMSA) | ATACTATGACGGGGTCGTTAA |
| MdPH5-F(IL60) | TTCGTCGACAAGCTTATGGCTGAAGATCTGGAGAAACC |
| MdPH5-R(IL60) | TAGCCTAGGCTCGAAGACTGTGTGGGCTGATT |
| MdPH5-F(TRV) | AGAAGGCCTCCATGGGGATCCCTCACCCTGAATCGCCTAA |
| MdPH5-R(TRV) | TGTCTTCGGGACATGCCCGGGGCATAGATCTAGAATCTGTT |

**Supplemental Table 2. *Cis*-elements analysis of *MdPH5* promoter regions.**

| *Cis*-Element Name | *Cis*-Element Sequence | Function | Start site (bp) | Termination sites (bp) |
| --- | --- | --- | --- | --- |
| MBS | CAACTG | MYB binding site involved in drought-inducibility | +828 | +834 |
| GT1-motif | GGTTAA | light responsive element | +377 | +383 |
| P-box | CCTTTTG | gibberellin-responsive element | -556 | -549 |
| G-Box | CACGTT | cis-acting regulatory element involved in light responsiveness | +292 | +298 |
| ABRE | ACGTG | cis-acting element involved in the abscisic acid responsiveness | -292 | -287 |
| Gap-box | CAAATGAA(A/G)A | part of a light responsive element | +268 | +278 |
| A-box | CCGTCC | cis-acting regulatory element | +244 | +251 |
| TC-rich repeats | ATTCTCTAAC | cis-acting element involved in defense and stress responsiveness | +46 | +55 |
| chs-Unit 1 m1 | ACCTAACCCGG | part of a light responsive element | -369 | -359 |
| ARE | AAACCA | cis-acting regulatory element essential for the anaerobic induction | +1167 | +1173 |
| GATA-motif | AAGGATAAGG | part of a light responsive element | -1268 | -1259 |
| CCAAT-box | CAACGG | MYBHv1 binding site | -801 | -795 |
| Box 4 | ATTAAT | part of a conserved DNA module involved in light responsiveness | +24 | +30 |
| TGACG-motif | TGACG | cis-acting regulatory element involved in the MeJA-responsiveness | +839 | +844 |
| Box II | CCACGTGGC | part of a light responsive element | -936 | -927 |
| TCA-element | CCATCTTTTT | cis-acting element involved in salicylic acid responsiveness | +1078 | +1087 |
| MSA-like | TCAAACGGT | cis-acting element involved in cell cycle regulation | +447 | +456 |
| TGA-element | AACGAC | auxin-responsive element | -857 | -851 |

**Supplemental Table 3. *Cis*-elements analysis of *MdMYB73* promoter regions.**

| *Cis*-Element Name | *Cis*-Element Sequence | Function | Start site (bp) | Termination sites (bp) |
| --- | --- | --- | --- | --- |
| ACE | CTAACGTATT | cis-acting element involved in light responsiveness | +909 | +918 |
| Box 4 | ATTAAT | part of a conserved DNA module involved in light responsiveness | +94 | +100 |
| ABRE | ACGTG | cis-acting element involved in the abscisic acid responsiveness | -142 | -137 |
| ARE | AAACCA | cis-acting regulatory element essential for the anaerobic induction | +289 | +295 |
| MBS | CAACTG | MYB binding site involved in drought-inducibility | -302 | -296 |
| GC-motif | CCCCCG | enhancer-like element involved in anoxic specific inducibility | -1785 | -1779 |
| WUN-motif | AAATTTCCT | wound-responsive element | +1333 | +1342 |
| Gap-box | CAAATGAA(A/G)A | part of a light responsive element | -696 | -687 |
| LTR | CCGAAA | cis-acting element involved in low-temperature responsiveness | +1795 | +1801 |
| GARE-motif | TCTGTTG | gibberellin-responsive element | +241 | +248 |
| CCAAT-box | CAACGG | MYBHv1 binding site | -629 | -623 |
| G-box | TACGTG | cis-acting regulatory element involved in light responsiveness | -142 | -136 |
